# Management of transitions to adult services for young people with eating disorders: survey of current practice in England

**DOI:** 10.1192/bjb.2021.109

**Published:** 2023-02

**Authors:** Anthony P. Winston, Samantha Child, Joseph Jackson, Moli Paul

**Affiliations:** 1 Aspen Centre, Coventry and Warwickshire Partnership Trust, Warwick, UK; 2 Stratford Child and Adolescent Mental Health Services, Coventry and Warwickshire Partnership Trust, Stratford-upon-Avon, UK; 3 Warwick Medical School, UK

**Keywords:** Carers, transition, eating disorders, service organisation, service users

## Abstract

**Aims and method:**

The Royal College of Psychiatrists has published recommendations for managing transitions between child and adolescent mental health services (CAMHS) and adult services for eating disorders. A self-report questionnaire was designed to establish how many CAMHS teams meet these recommendations and was distributed to 70 teams providing eating disorders treatment in England.

**Results:**

Of the 38 services that participated, 31 (81.6%) reported a flexible upper age limit for treatment. Only 6 services (15.8%) always transferred young people to a specialist adult eating disorders service and the majority transferred patients to either a specialist service or a community mental health team. Most services complied with recommended provision such as a written transition protocol (52.6%), individualised transition plans (78.9%), joint care with adult services (89.5%) and transition support for the family (73.7%).

**Clinical implications:**

Services are largely compliant with the recommendations. It is a concern that only a small proportion of services are always able to refer to a specialist adult service and this is likely to be due to a relative lack of investment in adult services.

Transitions between child and adolescent and adult services are recognised as problematic within health and social care^1^ and there is concern about discontinuity of care between child and adolescent mental health services (CAMHS) and adult mental health services.^2^ A substantial body of research indicates that discontinuity between CAMHS and adult services in general often leads to poor experiences of care, drop-out, unsuccessful treatment and potentially avoidable readmission to hospital.^3–7^ The NHS Long Term Plan emphasises the dangers of transitions between services for those aged 16–18 and acknowledges that ‘failure to achieve a safe transition can lead to disengagement, failure to take responsibility for their condition and ultimately poorer health outcomes’.^8^

Eating disorders are ‘prevalent, potentially lethal, and treatable’.^9^ In primary care, they are twice as common in people aged 16–20 than in those aged 11–15 or 21–24 years.^10^ Anorexia nervosa has a peak incidence in early to mid-adolescence^11,12^ and a duration of 3 years or more in 50% of cases.^13^ A study of first-episode anorexia nervosa in young people aged 8–17 years in contact with CAMHS in the UK and Ireland found that the mean age was 14.6 (s.d. = 1.66).^14^ Many individuals therefore present initially to CAMHS but are likely to need transfer on to adult services. Patients with eating disorders who are making the transition from CAMHS to adult services are likely to be particularly vulnerable to poor outcomes and effective management of transitions should therefore be a priority. The proportion of patients who are transferred to adult eating disorders services is not known but Arcelus et al^15^ found that 27.7% of those referred to an adult eating disorders service had previously been seen in CAMHS. Clinical experience suggests that a significant number of patients whose treatment is not complete when they reach the upper age threshold for CAMHS are unable to access adult eating disorders services; they may instead find themselves being treated in generic adult mental health services or discharged to primary care.

Eating disorders are associated with major longer-term burdens in terms of mortality and morbidity and people with eating disorders have a higher prevalence of other mental disorders compared with controls of the same gender and age.^16,17^ A 30-year outcome study indicates that 20% of those with adolescent-onset anorexia nervosa will develop a chronic condition.^18^

The transition from CAMHS to adult services takes place at a developmentally sensitive time, when the young person is searching for autonomy and identity. It is often associated with other transitions, such as leaving home to go to university, and with a change of therapeutic model between CAMHS and adult services.^19^ NHS England has issued guidance for the development of eating disorders services for children and young people,^20^ which recommends the commissioning of local community eating disorders services for children and young people (CEDS-CYP), with the necessary capacity and skills mix to meet the associated access and waiting-time standard. The Royal College of Psychiatrists (RCPsych) has published guidance on managing transitions to adult services for young people with an eating disorder,^21^ based on both clinical experience and the evidence about mental health transitions in general; the lead author of the present study is one of the authors of this guidance. The present study aimed to establish how well the recommendations in the RCPsych guidance are being met.

## Method

To help CEDS-CYP teams to meet the standards set out by NHS England,^20^ a series of national training days was provided, which were hosted by the RCPsych.

A self-report questionnaire was designed for the study by the research team, based on the RCPsych's guidance (a blank questionnaire is available in the supplementary material, available at https://dx.doi.org/10.1192/bjb.2021.109). The research team decided to administer the questionnaire at the national training days, as they were attended by the majority of CEDS-CYP teams in England. Teams were invited to complete the study questionnaire at the training days and a questionnaire was sent out by post to any teams not present at the training days. Participation in the study was entirely voluntary. Results were tabulated in Excel. As this study was a service evaluation and did not involve patients, research ethics approval was not required.

## Results

**Fig. 1:**
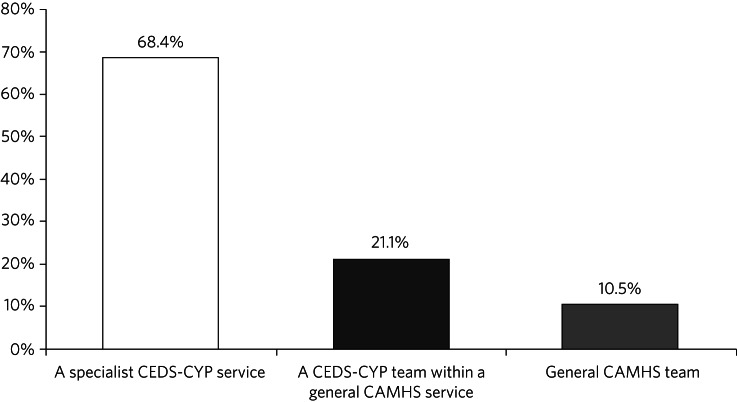
Breakdown by service type for 38 child and adolescent mental health services in England providing treatment for eating disorders. CEDS-CYP, community eating disorders services for children and young people; CAMHS, child and adolescent mental health services.

Forty-three out of seventy services (61%) responded (Fig. 1). Five of the services that responded were all-age services and were therefore excluded, giving a total of 38 that were included in the analysis. The overall response rate among eligible services was therefore 58%. Results are summarised in Fig. 1.

### Age at transition

Thirty-seven services (97.4%) had a fixed upper age limit for treatment (transition boundary) and one (2.6%) did not. Within those services with a fixed transition boundary, two (54%) set the limit at 16 years, twenty-five (67.6%) set it at 18 years, two (5.4%) at 18.5 years, seven (18.9%) at 19 years and one (2.7%) at 25 years. For twenty-nine services (78.4%) the transition boundary was the same as that at which the adult services take on patients but for nine (24.3%) it was not.

### Destination within adult services

Six services (15.8%) always transferred young people to a specialist adult eating disorders service. Twenty-eight services (73.7%) reported that some young people went to a specialist eating disorders service and some to a community mental health team. Three services (7.9%) reported that all young people were transferred to a community mental health team. Thirteen services (34.2%) answered ‘other’ to this question and several services selected more than one answer. Destinations for patients in the ‘other’ category included general practitioner, the Improving Access to Psychological Therapies programme and the voluntary sector. Twenty-one services (55.3%) reported that young people from their service are sometimes discharged when they reach transition age because they do not meet the access criteria for adult services. Four services (10.5%) did not answer this question.

### Transition protocols and planning

**Fig. 2:**
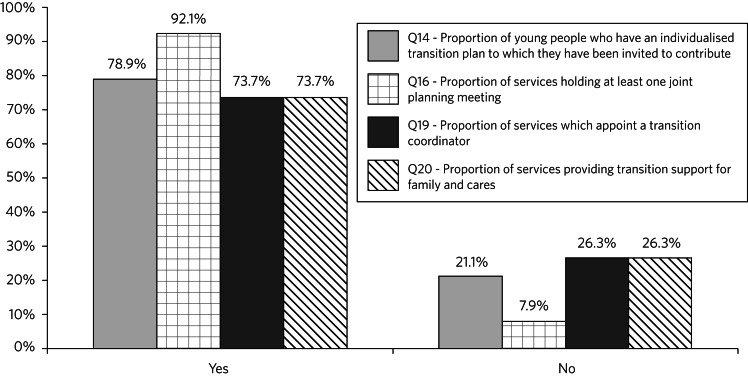
Transition planning arrangements for 38 child and adolescent mental health services in England providing treatment for eating disorders.

Twenty services (52.6%) had a transition protocol specifically for young people with eating disorders and twenty-six (68.4%) also had a generic protocol for all young people making the transition to adult services. Thirty-four services (89.5%) had a procedure for identifying young people who would be moving from CAMHS to adult services at least 6 months before the planned transition. Twenty-eight services (73.7%) routinely had active discussions with the adult service, beginning at least 6 months before the planned transition. Twenty-two services (57.9%) reported involving paediatric/medical services, twenty-one (53.3%) social care, nineteen (50%) education and twenty (52.6%) the general practitioner (GP) during transition planning. Further details of transition planning arrangements are shown in Fig. 2.

### Therapeutic model

**Fig. 3:**
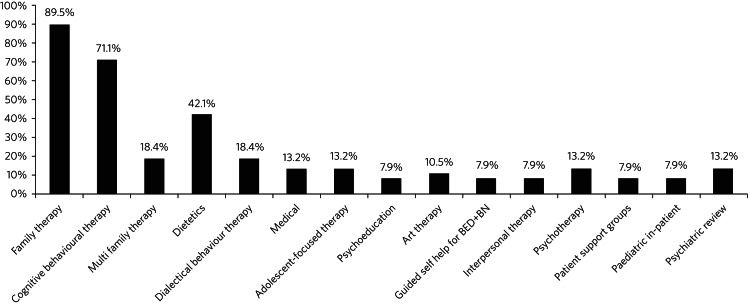
Therapeutic interventions offered by 38 child and adolescent metal health services in England providing treatment for eating disorders. BED+BN, binge eating disorder and bulimia nervosa.

Services offered a wide variety of therapeutic interventions (Fig. 3). Thirty-seven services (97.4%) reported that there was a difference in therapeutic model/orientation between themselves and adult services. Twenty-one services (55.3%) commented on CAMHS having a more family-focused approach, whereas adult services were more focused on the individual. Twenty-seven services (71.1%) reported that their transition planning addressed changes in the therapeutic model, but six services (15.8%) did not answer this question. Twenty-seven services (71.1%) reported having a good understanding of how the adult services to which they refer operated. One service commented on the big difference in the risk threshold between CAMHS and adult services. Another commented that the ‘adult system is based around BMI criteria which not all of our young people fit into’.

### Joint working

Thirty-four services (89.5%) reported having a period of joint working between CAMHS and adult services prior to transfer.

### Transition timing

Thirty-one services (81.6%) reported that the timing of transition was flexible and based on the young person's needs. Twenty-nine (76.3%) reported that they could delay the time of transition and nineteen (50%) said they could bring it forward if the young person was in crisis. Twenty services (52.6%) reported that the time of transition was influenced by the availability of specific therapies in either service. Nine services (23.7%) reported that the start of treatment for newly referred young people was sometimes delayed because the young person was approaching transition age.

### Clinical responsibility

Twenty-five services (65.8%) reported that CAMHS held clinical responsibility during the period of joint working, four (10.5%) that adult services held responsibility and nine (23.7%) that it was shared. In response to the question ‘Is medical responsibility ever held in one service while clinical intervention is provided by another?’, eight services (21.1%) answered ‘yes’ and twenty-four (63.2%) answered ‘no’; six (15.8%) did not answer this question. For the services that answered ‘yes’, explanations given included: ‘if the patient is reaching 18 they are invited to an RO-DBT [radically open dialectical behaviour therapy] group in the adult service’; ‘sometimes the adult ED [eating disorder] service does the work whilst CAMHS holds psychiatric/medical responsibility’; ‘consultant works across both teams’; ‘the adult service takes medical responsibility but family therapy in CAMHS continues for a brief period of time’; and ‘if adult services have a young adult who wishes to access groups’.

### Documentation

Twenty-five services (65.8%) reported that they had electronic records systems that supported joint working between CAMHS and adult services and one did not answer this question. Thirty-five (92.1%) reported that they shared a summary of the case history with adult services when transfer of care was requested; the same number reported that they shared risk assessments and twenty-one (55.3%) said that they shared other information. Eight services (21.1%) reported that they had a shared electronic records system so all documentation was shared.

## Discussion

This study surveyed a large number of services in England which provide treatment for children and adolescents with eating disorders. It is of note that the majority of services were CEDS-CYP, rather than generic CAMHS, reflecting the recent increase in investment in eating disorder services for this group. The results of the survey are broadly encouraging and suggest that for the most part the recommendations in the RCPsych's guidelines^21^ are being followed.

Although the majority of services reported having a fixed upper age for treatment, many also reported that the timing of transition was flexible and based on the young person's needs. The most likely explanation for these apparently inconsistent findings is that services have an age at which transition is expected but that this can be modified in the light of individual circumstances. Just over half of the services contacted reported having a written protocol for managing transitions.

CAMHS teams in England appear to be doing reasonably well in terms of identifying well in advance young people who are likely to need transfer and working with adult services to manage the transfer; the majority of services had a period of joint working prior to transfer. However, the survey found some important gaps in provision in some services: 21.1% of services did not use individualised transition plans and 26.3% failed to provide transition support for the family and carers. Only 73.7% provided a transition coordinator to support the young person through the transition. It is to be hoped that wider dissemination of the RCPsych guidelines, supported by appropriate training, will lead to further improvement in these areas.

Coordinated care is very difficult when there is a break between services, so it is a concern that 23.7% of services reported a discrepancy between the age at which CAMHS stopped and adult services began. This is likely to increase significantly the risk of young people dropping out of contact with services. This fragmentation of services also makes it impossible to implement interventions such as First Episode Rapid Early Intervention for Eating Disorders (FREED),^22,23^ which span both CAMHS and adult services. The fact that in 7.9% of services all young people go to a community mental health team presumably indicates that some areas of the country still lack any specialist eating disorders services for adults.

Services offered a wide variety of therapeutic interventions. It is possible that this makes it difficult to ensure consistency of therapeutic approach when the young person makes the transition to adult services. Almost all services reported a difference in therapeutic orientation between the CAMHS and the adult service. As expected, the principal difference was frequently that the CAMHS provided a more family-focused approach, whereas the adult service was more individually focused; this may well be developmentally appropriate. Despite these concerns, only 71.1% of services addressed the issue of differences in therapeutic model during transition planning and this clearly needs to be improved.

One of the most worrying findings was that only a minority of services reported that young people are always transferred to a specialist adult eating disorders service. The majority reported that some young people go to a specialist service whereas others go to generic services, presumably on the basis of the perceived severity of their eating disorder. Although some young people may not need a specialist adult service because their needs are better met elsewhere, the most likely explanation for this finding is that adult eating disorders services are unable to take them owing to more stringent access criteria, which are driven by resource constraints. This explanation is supported by the finding that more than half of the services reported that young people are sometimes discharged from treatment because they do not meet the referral criteria for local adult services.

Patients who do not transfer to a specialist adult eating disorders service are likely to be managed within generic community mental health teams. These teams often lack training and skills in the management of eating disorders and outcomes are likely to be inferior as a result. The disparity in service provision between CEDS-CYP and adult eating disorders services reflects the fact that, in 2014, an additional £30 million per year was allocated to support the development of CEDS-CYP in England. However, at the time of the study, there had been no comparable new investment in adult eating disorder services.

Since the study was completed, however, NHS England and NHS Improvement have announced a Community Mental Health Transformation Framework for Adults and Older Adults, which forms part of the NHS Long Term Plan.^24^ This programme will deliver substantial new investment in community eating disorders services and it is to be hoped that it will result in parity of provision across the age range. Both the NHS Long Term Plan and the Community Mental Health Framework for Adults and Older Adults stress the importance of improving transitions from CYP to adult services. This new funding will offer an opportunity to embed the principles of good transition management in the design of services and address some of the shortfalls identified in this study.

### Limitations

The study has a number of limitations. It covered only services operating in England and it would be of interest to discover whether the results are similar in other parts of the UK. The response rate of 58% could be a source of bias, as services that do not manage transitions well may have been less willing to return the questionnaires. Unfortunately, the study design did not allow us to obtain data on the non-responder services.

Importantly, the survey was based on self-reporting by the services involved and did not assess service delivery independently. It focused on CAMHS as they are responsible for initiating transitions. It would have been informative to have data on the types of intervention offered by adult services but such data would have been difficult to obtain as, at the time of the study, there was no comprehensive list of adult eating disorders services available. This study was based on reports from services themselves of what they provide. However, future research could usefully explore the patient experience and how closely this corresponds to what services believe themselves to be providing. Finally, the survey only sought information from professionals and future qualitative work could usefully explore the patient and carer experience of transitions.

### Recommendations

The results of this study suggest a number of recommendations for improving age-related transitions for patients with eating disorders. All services should have written transition protocols; these would undoubtedly benefit from co-production with patients and their families. Provision of a transition coordinator is established as a valuable intervention and should be standard in all services. Providers and commissioners need to give greater attention to aligning CEDS-CYP and adult eating disorders services, ensuring that access criteria are consistent and that there is no age gap between them. At the same time, clinicians should pay more attention to helping patients and families to negotiate the differences in therapeutic approach between CEDS-CYP and adult services.

## Data Availability

Study data are available on request from the first author A.P.W..

## About the authors

**Anthony P. Winston** is a consultant in eating disorders at the Aspen Centre, Coventry and Warwickshire Partnership Trust, Warwick, UK. When this study was carried out **Samantha Child** was a Core Trainee Year 3 (CT3) in psychiatry and **Joseph Jackson** was a Specialty Trainee Year 4 (ST4) in psychiatry at the Aspen Centre, Coventry and Warwickshire Partnership Trust, Warwick. **Moli Paul** was a consultant child and adolescent psychiatrist with Stratford Child and Adolescent Mental Health Services, Coventry and Warwickshire Partnership Trust, and an Honorary Associate Clinical Professor in the Division of Health Sciences, Warwick Medical School, UK.

## Supplementary material

For supplementary material accompanying this paper visit https://doi.org/10.1192/bjb.2021.109. click here to view supplementary material

## References

[1] National Institute for Health and Care Excellence. Transition from Children's to Adults’ Services for Young People Using Health or Social Care Services. NICE Guideline: Full Version. NICE, 2016 (https://www.nice.org.uk/guidance/ng43/evidence/full-guideline-2360240173).

[2] Signorini G, Singh SP, Marsanic VB, Dieleman G, Dodig-Ćurković K, Franic T, The interface between child/adolescent and adult mental health services: results from a European 28-country survey. Eur Child Adolesc Psychiatry 2018; 27: 501–11. 29368253 10.1007/s00787-018-1112-5

[3] National CAMHS Review. Children and Young People in Mind: The Final Report of the National CAMHS Review. Child and Adolescent Mental Health Services, 2008.

[4] Lamb C, Hall D, Kelvin R, Van Beinum M. Working at the CAMHS/Adult Interface: Good Practice Guidance for the Provision of Psychiatric Services to Adolescents/Young Adults. *A* Joint Paper *from the Interfaculty Working Group of the Child and Adolescent Faculty and the General and Community Faculty of the Royal College of Psychiatrists*. Royal College of Psychiatrists, 2008.

[5] Singh SP. Transition of care from child to adult mental health services: the great divide. Curr Opin Psychiatry 2009; 22: 386–90. 19417667 10.1097/YCO.0b013e32832c9221

[6] Singh SP, Paul M, Ford T, Kramer T, Weaver T, McLaren S, Process, outcome and experience of transition from child to adult mental healthcare: multiperspective study. Br J Psychiatry 2010; 197: 305–12. 20884954 10.1192/bjp.bp.109.075135

[7] Muñoz-Solomando A, Townley M, Williams R. Improving transitions for young people who move from child and adolescent mental health services to mental health services for adults: lessons from research and young people's and practitioners’ experiences. Curr Opin Psychiatry 2010; 23: 311–7. 20520550 10.1097/YCO.0b013e32833a51e2

[8] NHS England. The NHS Long Term Plan. NHS, 2019 (https://www.longtermplan.nhs.uk/wp-content/uploads/2019/08/nhs-long-term-plan-version-1.2.pdf)

[9] Nicholls D, Becker A. Food for thought: bringing eating disorders out of the shadows. Br J Psychiatry 2020; 216: 67–8. 31347478 10.1192/bjp.2019.179

[10] Wood S, Marchant A, Allsopp M, Wilkinson K, Bethel J, Jones H, Epidemiology of eating disorders in primary care in children and young people: a clinical practice research datalink study in England. BMJ Open 2019; 9(8): e026691. 10.1136/bmjopen-2018-026691 PMC6688704 31378721

[11] Kohn M, Golden NH. Eating disorders in children and adolescents: epidemiology, diagnosis and treatment. Paediatr Drugs 2001; 3: 91–9. 11269642 10.2165/00128072-200103020-00002

[12] Micali N, Hagberg KW, Petersen I, Treasure JL. The incidence of eating disorders in the UK in 2000–2009: findings from the general practice research database. BMJ Open 2013; 3(5): e002646. 10.1136/bmjopen-2013-002646 PMC3657659 23793681

[13] Keski-Rahkonen A, Hoek HW, Susser ES, Linna MS, Sihvola E, Raevuori A, Epidemiology and course of anorexia nervosa in the community. Am J Psychiatry 2007; 164: 1259–65. 17671290 10.1176/appi.ajp.2007.06081388

[14] Petkova H, Simic M, Nicholls D, Ford T, Prina AM, Stuart R, Incidence of anorexia nervosa in young people in the UK and Ireland: a national surveillance study. BMJ Open 2019; 9(10): e027339. 10.1136/bmjopen-2018-027339 PMC6954494 31640991

[15] Arcelus J, Bouman WP, Morgan JF. Treating young people with eating disorders: transition from child mental health to specialist adult eating disorder services. Eur Eat Disord Rev 2008; 16: 30–6. 17910032 10.1002/erv.830

[16] Keski-Rahkonen A, Mustelin L. Epidemiology of eating disorders in Europe: prevalence, incidence, comorbidity, course, consequences, and risk factors. Curr Opin Psychiatry 2016; 29: 340–5. 27662598 10.1097/YCO.0000000000000278

[17] Demmler JC, Brophy ST, Marchant A, John A, Tan JOA. Shining the light on eating disorders, incidence, prognosis and profiling of patients in primary and secondary care: national data linkage study. Br J Psychiatry 2020; 216: 105–12. 31256764 10.1192/bjp.2019.153 PMC7557634

[18] Dobrescu SR, Dinkler L, Gillberg C, Råstam M, Wentz E. Anorexia nervosa: 30-year outcome. Br J Psychiatry 2020; 216(2): 97–104. 31113504 10.1192/bjp.2019.113 PMC7557598

[19] Winston AP, Paul M, Juanola-Borrat Y. The same but different? Treatment of anorexia nervosa in adolescents and adults. Eur Eat Disord Rev 2012; 20: 89–93. 21913286 10.1002/erv.1137

[20] NHS England. Access and Waiting Time Standard for Children and Young People with an Eating Disorder: Commissioning Guide. National Collaborating Centre for Mental Health, 2015.

[21] Royal College of Psychiatrists. Managing Transitions When the Patient Has an Eating Disorder: Guidance for Good Practice (College Report CR208). RCPsych, 2017.

[22] Brown A, McClelland J, Boysen E, Mountford V, Glennon D, Schmidt U. The FREED project (First Episode and Rapid Early Intervention in Eating Disorders): service model, feasibility and acceptability. Early Interv Psychiatry 2018; 12: 250–7. 27619198 10.1111/eip.12382

[23] McClelland J, Hodsoll J, Brown A, Lang K, Boysen E, Flynn M, A pilot evaluation of a novel first episode and rapid early intervention service for eating disorders (FREED). Eur Eat Disord Rev 2018; 26: 129–40. 29460477 10.1002/erv.2579

[24] NHS England, NHS Improvement, National Collaborating Central for Mental Health. The Community Mental Health Framework for Adults and Older Adults. NHS England, 2019.

