# Supplementary material for: Management of transitions to adult services for young people with eating disorders: survey of current practice in England
Source: BJPsych Bull. 2023 Feb;47(1):17–22. doi: 10.1192/bjb.2021.109 (PMC10028553; doi:10.1192/bjb.2021.109)
Supplement: Supplementary file 1 [file S2056469421001091sup001.docx]

TRANSITIONS FROM COMMUNITY CAMHS TO ADULT SERVICES FOR YOUNG PEOPLE WITH EATING DISORDERS

QUESTIONNAIRE

Name of service……………………………………………………………………………………………………………………………..

Address……………………………………………………………………………………………………………………………………………

Phone number…………………………………………………………………………

Name of person completing form……………………………………………………………………………………………………..

Designation………………………………………………………………………………………..

Email………………………………………………………………………………………..

**ABOUT YOUR SERVICE**

Do you work in:

1. A specialist CAMHS eating disorders service? 🞎
2. A Children and Young People’s Eating Disorder Team within a

specialist CAMHS team? 🞎

1. A generic specialist CAMHS team? 🞎

Is there a fixed upper age limit for treatment (transition boundary) in your service? YES 🞎 NO 🞎

If so, what is it?...............

When young people with eating disorders are transferred from your service, where do they go?

1. All go to a specialist adult eating disorders service 🞎
2. Some go to a specialist eating disorders service and some to a

community mental health team 🞎

1. All go to a community mental health team 🞎
2. Other ………………………………………………………….

What therapies/interventions are available for young people with eating disorders in your service?

...............................................................................................................................................................

……………………………………………………………………………………………………………………………………………………………

Does your service have a written protocol specifically for young people

with eating disorders who are making the transition to adult services? YES 🞎 NO 🞎

Does your service have a generic written protocol for young people

making the transition to adult services? YES 🞎 NO 🞎

Please specify which adult care providers are specified in the protocol:

...............................................................................................................................................................

……………………………………………………………………………………………………………………………………………………………

Is the age at which young people are discharged from your service

(transition boundary) the same as that at which they are taken on

by adult services? YES 🞎 NO 🞎

**TRANSITION PLANNING**

Does your service have a procedure for identifying young people who will

be moving from CAMHS to adult services at least six months before the

planned transition? YES 🞎 NO 🞎

If yes, is the appropriate adult service contacted routinely at this point? YES 🞎 NO 🞎

Does active discussion with the adult service routinely begin at least

six months before the planned transition? YES 🞎 NO 🞎

Which of the flowing services are involved in transition planning if they have been significantly involved in the young person’s care?

(a) Paediatric/medical services 🞎

(b) Social care 🞎

(c) Education 🞎

(d) GP 🞎

(e) Other……………………………………………………………………

Do young people have an individualised transition plan to which

they have been invited to contribute? YES 🞎 NO 🞎

Do you have a standardised format for recording the individualised

transition plan? YES 🞎 NO 🞎

Does your service hold at least one joint discharge planning meeting,

involving both services, before discharge from CAMHS? YES 🞎 NO 🞎

Does this joint planning meeting include the patient? YES 🞎 NO 🞎

Does this meeting include the family or carers? YES 🞎 NO 🞎

Is a transition coordinator/ named worker appointed to support the

young person through the period of transition? YES 🞎 NO 🞎

Is transition support also provided for the family and carers YES 🞎 NO 🞎

If so what?

……………………………………………………………………………………………………………………………………………………

Is there a difference in therapeutic model/orientation between that

used in CAMHS and that used in adult services? YES 🞎 NO 🞎

If so, what are the models/orientations?

……............................................................................................................................................................

……………………………………………………………………………………………………………………………………………………………

Does transition planning address changes in therapeutic model

between that used in CAMHS and that used in adult services?? YES 🞎 NO 🞎

**TRANSITION TIMING**

Is the timing of transition flexible, taking into account the young

person’s degree of maturity and separation from family, the need

for ongoing work with the family/carers, education and employment

issues, links with other medical and social services and the views

of the patient and carers? YES 🞎 NO 🞎

Can the time of transition be delayed or brought forward if the young person is in crisis?

(a) Delayed 🞎

(b) Brought forward 🞎

(c) Neither 🞎

Is the timing of transition influenced by the availability of

specific therapies in either service? YES 🞎 NO 🞎

For newly referred young people, is the start of treatment within

your service ever delayed because the young person is approaching

transition age? YES 🞎 NO 🞎

**JOINT WORKING**

Is there a period of joint working between CAMHS and adult services

prior to transfer for young people who are already in your service? YES 🞎 NO 🞎

Does joint working involve

1. Joint planning of the transition? 🞎
2. Preparation for transition by staff from the adult service? 🞎
3. Therapy involving clinicians from both services ? 🞎
4. Other?………………………………………………….

Who holds clinical responsibility during the period of joint working?

1. CAMHS 🞎
2. Adult service 🞎
3. Shared between CAMHS and adult services 🞎

Is medical responsibility ever held in one service while clinical

intervention is provided by another? YES 🞎 NO 🞎

If yes, please specify:

...............................................................................................................................................................

……………………………………………………………………………………………………………………………………………………………

**OTHER ISSUES**

Do you feel you have a good understanding of how the adult

services to which you refer operate? YES 🞎 NO 🞎

Do your electronic systems support joint working between

CAMHS and adult services? YES 🞎 NO 🞎

What documentation is shared with adult services when transfer of care is requested?

Summary of case history? 🞎

Risk assessment? 🞎

Other? Please specify

...............................................................................................................................................................

……………………………………………………………………………………………………………………………………………………………

Are young people from your service ever discharged when they reach

transition age because they do not meet the access criteria for adult services? YES 🞎 NO 🞎

Is there anything else you would like to say about transitions for young people with eating disorders?

……………………………………………………………………………………………………………………………………………………………

……………………………………………………………………………………………………………………………………………………………

……………………………………………………………………………………………………………………………………………………………

…………………………………………………………………………………………………………………………………………………………….

**Thank you for your help**
